# Contrast-enhanced ultrasonography diagnosis of fundal localized type of gallbladder adenomyomatosis

**DOI:** 10.1186/s12876-015-0326-y

**Published:** 2015-08-05

**Authors:** ShaoShan Tang, LiPing Huang, Yao Wang, YiJiao Wang

**Affiliations:** Department of ultrasound, Shengjing hospital of China Medical University, No. 36 Sanhao Street, Heping District, Shenyang, 110004 China

## Abstract

**Background:**

Adenomyomatosis of gallbladder is an acquired hyperplastic lesion, characterized by focal or diffuse thickening of the gallbladder with intramural cysts or echogenic areas with comet tail on ultrasonography. But in some cases, especially in the localized fundal type of adenomyomatosis, the intramural anechoic cystic spaces are uncertainty which causes difficult to differential adenomyomatosis from GB cancer. The purpose of this study was to determine the accuracy of real-time contrast-enhanced ultrasonography(CEUS) in the diagnosis of the fundal localized type of gallbladder adenomyomatosis.

**Methods:**

We performed a retrospective study of 21 patients with pathologically proven fundal localized type of gallbladder (GB) adenomyomatosis. All patients underwent preoperative grayscale ultrasound (US) and real-time CEUS examination. The study’s reviewers made the diagnosis of adenomyomatosis according to the presence of the focal thickening of the fundal gallbladder wall with intramural cyst or intramural echogenic foci on grayscale US or CEUS. The diagnostic accuracy of US and CEUS was compared. The enhanced pattern and degree of intactness of the GB wall were also recorded.

**Results:**

The fundal portion of the GB wall showed localized thickness in all 21 patients. Small anechoic spaces or intramural echogenic foci were detected in 14 (66.7 %) and 21 (100 %) of cases respectively, and the intactness of the GB wall’s outer hyper-echoic layer was demonstrated in 17 (81 %) and 20 (95 %) on grayscale US and CEUS, respectively. The accuracy rate of the above two examination modalities was significantly different (*p* < 0.05). In the arterial phase of the CEUS, areas of focal thickened GB wall were iso-enhanced in 18 cases and hyper-enhanced in 3 cases. All 21 cases appeared to show heterogeneous enhancement with small non-enhancement spaces. The mucosal and serosal layers of the GB wall surrounding the lesions were enhanced, which presented as two “hyper-echoic lines” in the arterial phase of CEUS. In the venous phase of the CEUS, 19 lesions were iso-enhanced and 2 lesions were hypo-enhanced. The small non-enhancement spaces were more clearly during the venous phase.

**Conclusion:**

The small non-enhancement space is a characteristic finding of the fundal localized type of gallbladder adenomyomatosis on CEUS. CEUS could increase the degree of visualization of Rokitansky-Aschoff sinuses (RAS) and intactness of the GB wall, which play an important role in differential diagnosis.

## Background

Adenomyomatosis of the gallbladder is characterized by hyperplastic changes in the mucosal layer, with the formation of intramural diverticula or sinus tracts termed Rokitansky-Aschoff sinuses penetrating into a thickened muscular layer. Adenomyomatosis is commonly detected in 2 %-5 % of all cholecystectomies [1–3]. In most patients, the adenomyomatosis is asymptomatic but is detected by an incidental US examination or by post-operative examination of gallbladder specimens. US examination is the first line investigation used to diagnose adenomyomatosis of the GB. The criteria for diagnosis of adenomyomatosis by ultrasonography are localized or diffuse thickening of the GB wall and the presence of small intramural cyst(s) with or without intramural echogenic foci [4–8]. But some cases are misdiagnosed as chronic cholecystitis or cancer, because of the uncertainty of intramural anechoic cystic spaces. Thus, the diagnostic accuracy of ultrasonography in demonstrating adenomyomatosis has been reported as being less than 70 %, especially in the localized fundal type, which is the most common type of GB adenomyomatosis [9].

Recently, contrast-enhanced ultrasound (CEUS) has been used for the diagnosis of GB and biliary duct diseases and is considered a valuable complement to conventional US [10]. Some researchers have concluded that CEUS may be useful for differentiating malignant tumors from sludge, and could also differentiate cystic structures from parenchymal structures by dynamic observation of the blood-perfusion in the tumor or organ [11–13]. Therefore we propose that CEUS, by depicting the non-enhancing intramural diverticula could be helpful in detecting the tiny Rokitansky-Aschoff sinuses that are uncertain on grayscale US. In this paper,we present findings in the CEUS imaging of the fundal localized type of adenomyomatosis of the GB. To our knowledge, this is the first report on the use of CEUS in describing the fundal localized type of GB adenomyomatosis.

## Methods

Between December 2008 and November 2012, 258 patients with GB diseases received grayscale US and CEUS examinations and underwent cholecystectomy in our department. Of these, 21 patients were found to have fundal localized GB adenomyomatosis confirmed by the results of pathology testing. These 21 patients included 11 females and 10 males. Their ages ranged from 29 to 70 years old, average age 48.55 ± 11.27. Five patients had associated cholelithiasis causing right upper quadrant pain, 8 patients experienced discomfort in the right upper quadrant, while the other 8 patients were asymptomatic but were detected incidentally on routine US examination. All patients underwent laparoscopic cholecystectomy: due to cholelithiasis in 10 cases, GB polyps in 7 and GB wall thickening in 4 cases.

The US contrast agent was SonoVue (Brocca SpA, Milan, Italy), which consisted of stabilized sulphur hexafluoride microbubbles. A 2.4 ml bolus injection of SonoVue was administered via an antecubital vein, immediately followed by a 5-ml bolus of saline solution. CEUS was performed with GE LOGIQ-9 (GE Health-care, Milwaukee, WI), Toshiba APLIO (Toshiba Medical System, Tokyo, Japan) and a convex transducer with a central frequency of 2.5 MHz. The mechanical index of CEUS was set up at low level (MI0.08-0.12). The procedures were conducted by two investigators (SS. T, LP. H), both with at least 19-year experience in performing US examinations and more than 5-year experience in CEUS examinations. The real-time CEUS images were recorded on hard disc as soon as the contrast agent was injected and the time was calculated simultaneously. The entire examination was observed for 3 min. The enhancement process was divided into the arterial phase (0 to 30 s after contrast injection), and the venous phase (31 s after contrast injection) [14].

## Ethical approval of the study protocol

The study protocol received the approval from the ethics committee of China Medical University Shengjing Hospital (Shenyang, China). The informed consent of the study was obtained from all patients.

## Image analysis

Two examiners (SS. T,Y. W) independently analyzed the US images on a PACS workstation and CEUS real-time images on hard disc, with consensus. They were unaware of the clinical dates, other imaging findings, and the surgical results.

The reviewers recorded the following criteria: thickness, intramural cysts with or without intramural echogenic foci and intactness of the GB wall on US and CEUS, respectively. On CEUS, the enhancement pattern was described as homogeneous and interogeneous. The enhancement extent was divided into hyper-, iso-, hypo-, or non-enhancement, when compared with the normal GB wall during the arterial phase and venous phase.

## Statistical analysis

All data were analyzed by SPSS Statistics 17.0. The measurement data used in analysis were presented as mean ± standard deviation ($\overline{\mathrm{X}}\kern0.5em \pm \kern0.5em \mathrm{S}$). The comparison between the qualitative data was done using the Chi-square test. Statistical significance was defined as *P* values less than 0.05.

## Results

### Grayscale US

**Fig. 1:**
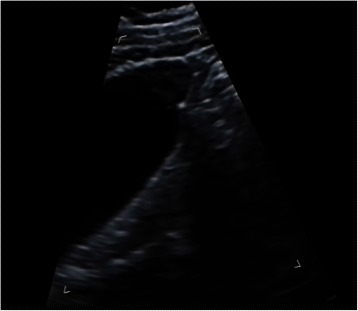
US image of fundal localized type GB adenomyomatousis. Localized thickness of GB wall in form of triangle at the fundus with small anechoic areas was detected

The GB walls were localized thickenings protruding into the lumen in form of triangles at the fundus in all patients. The thickness range was from 1.0 × 0.9 cm^2^ to 2.7 × 1.3 cm^2^. In 14 patients, small anechoic areas or intramural echogenic foci were detected in areas of thickened GB wall (Fig. 1). These 14 patients were diagnosed with certainty as adenomyomatosis. The thickened GB revealed a heterogeneous hypoechoic mass in 7 patients without small anechoic areas or intramural echogenic foci. Three of these 7 patients were suspected as having adenomyomatosis, due to two hyperechoic layers being observed around the lesions, which indicated the mucosa and serosa of the GB wall. The other 4 patients could not be excluded from a diagnosis of carcinoma because their local GB walls were obscured.

### CEUS

**Fig. 2:**
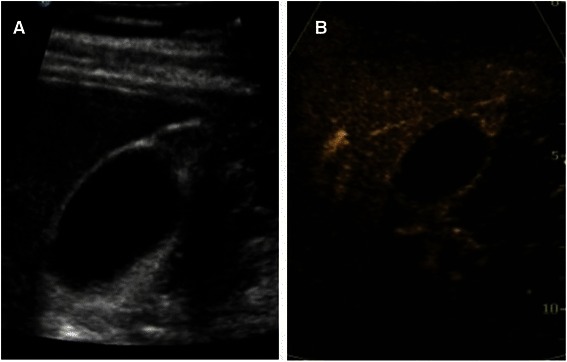
Image of US and CEUS. **a** The hypoechoic area was detected at the GB fundus on US. No anechoic area was showed but the GB wall was intactness. **b** CEUS—arterial phase (24 s)—heterogeneous enhancement with small anechoic spaces. The mucosa and serosa layer around the lesion was clear and intact

**Fig. 3:**
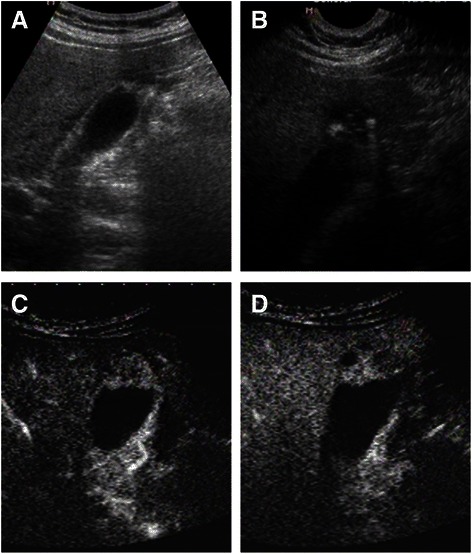
Image of US and CEUS. **a** The fundus of GB wall was thickened and the GB wall was obscure. **b** The intramural echogenic foci were detected by high frequency transducer. **c** CEUS—arterial phase (22 s) —heterogeneous hyper-enhancement and wall was intact. **d** CEUS—venous phase (34 s) the anechoic spaces were more clear

On CEUS, the lesions were iso-enhanced in 18 cases and hyper-enhanced in 3 cases during the arterial phase. All these 21 cases appeared as heterogeneous enhancement with a few small anechoic spaces (Fig. 2). The mucosal and serosal layers of thickened GB wall were enhanced at the arterial phase presenting as two “hyper-echoic lines” and were intactness in 20 cases (Fig. 3). The serosal layer showed discontinuity in 1 case. During the venous phase of CEUS, 19 lesions were iso-enhanced and 2 lesions were hypo-enhanced. The small non-enhancement spaces were more clearly during the venous phase.

All 21 patients underwent laparoscopic cholecystectomies. Histopathological examination confirmed the diagnosis of adenomyomatosis with chronic cholecystitis. One of 21 cases had malignant transformation with serosal layer invasion, in which the serosal layer showed discontinuity on CEUS.

The accuracy rates of the diagnosis of adenomyomatosis, made according to the presence of the focal thickening of fundal gallbladder wall with intramural cyst or intramural echogenic foci, were 66.7 % and 100 % on grayscale US and CEUS, respectively. The *P* value was less than 0.05, which indicates a statistically significant difference.

## Discussion

Adenomyomatosis is an acquired and degenerative disease of the gallbladder, mostly seen in adults. Adenomyomatosis consists of three types: localized, segmental and diffuse [15]. The localized type is the most common and usually involves the GB fundus. The fundal area of the GB is a superficial area where ultrasonography is more limited due to reverberation artifacts from subcutaneous tissue decreasing spatial resolution and interruption from gastrointestinal gas. Therefore, recognition of Rokitansky-Aschoff sinuses is more difficult at this part of the GB wall. But without the observation of intramural cysts, focal wall thickening is a non-specific finding; it may represent an early state of gallbladder carcinoma, chronic cholecystitis or localized adenomyomatosis [16]. Thus, recognition of intramural cysts is essential in the sonographic diagnosis of adenomyomatosis [8].

Microbubble US contrast agents are truly blood pool contrast agents. The agent remains entirely intravascular and with it, any abnormal vascular supply will demonstrate different enhancement. This property is of benefit in distinguishing tumors of the GB wall from pseudotumors; motionless sludge or debris will not be enhanced. The contrast enhancement could also increase spatial resolution and reduce artifact and noise. Rokitansky-Aschoff sinuses could be more clearly visualized as background GB wall enhances, because Rokitansky-Aschoff sinuses remains anechoic on CEUS [10]. Therefore, CEUS is better than US at helping to depict the intramural cysts of adenomyomatosis and making the correct diagnosis. In this study, only 66.7 % of cases had small anechoic areas in thickened GB wall on US, but 100 % cases had small no-enhancement areas on CEUS.

Adenomyomatosis is characterized by a thickened muscularis with Rokitansky-Aschoff sinuses, with the mucosal layer and serosal layer of thickened GB wall separate and intact. Ijin Joo et al. reported that the presence of a “multilayer pattern” in the GB wall, with a smooth innermost hyper-echoic layer, was useful in the diagnosis of adenomyomatosis, in cases where the anechoic area or intramural echogenic foci in thickened GB wall were not detected [17]. In this study, 3 cases of suspected adenomyomatosis on US before operation were observed because of the intact mucosal and serosal layers presenting as “multilayer pattern” of local thickened GB wall. But if the local GB wall thickness and intactness was obscured, the presence of a malign lesion could not be excluded, which occurred in 4 cases in our research. But in 3 of these 4 cases, the GB wall intactness was detected by CEUS and confirmed by surgery, due to the GB wall being enhanced at the early arterial phase and appearing as a “hyper-echoic line” compared with the surrounding hepatic parenchyma, which increases the depiction of the GB wall. CEUS was therefore more accurate for the detection of the intactness of the GB wall [18].

Although adenomyomatosis rarely has malignant potential, Nabatame et al. reported that adenomyomatosis in the fundal region of the GB in elderly patients has a higher risk of developing into GB carcinoma [19]. In this study, 1 patient with adenomyomatosis had malignant transformation and serosal layer invasion. The patient was seventy-two years old, and male. Conventional US and CECT suspected this patient having GB carcinoma but adenomyomatosis because of the obscure GB wall without intramural diverticulum. CEUS demonstrated the presence of non-enhancing areas suggestive of Rokitansky-Aschoff sinuses and the discontinuity of the wall. These CEUS findings may be helpful in arriving at the diagnosis of adenomyomatosis with malignant transformation.

The studies of CEUS have been concluded that the second-generation contrast agents could improve the diagnostic accuracy in GB diseases. Namata K et al [20]. reported that tumor vascular pattern on CEUS was useful for differentiating GB cancer from benign polypoid diseases. The branched type and tortuous type tumor vessels were considered as indicative of cancer and had a high accuracy [12, 20]. The hyper-enhanced in the arterial phase and washout in the venous phase of CEUS is not applicable to diagnose GB malignant disease, but the contrast agent washout time for malignant and benign GB disease presented significant difference [20, 21]. UP to now, researches of CEUS for GB diseases concentrated on differential malignant from benign in general rather than a kind of special disease or including only a small number of this subject. In this study, to further estimate CEUS is helpful in diagnosis of GB local wall thickening, 21 fundal localized type of GB adenomyomatosis were evaluated. A few small anechoic spaces observed in both arterial and venous phase was characteristic finding of adenomyomatosis. The mucosal and serosal layers were hyper-enhanced at the arterial phase presenting as two “hyper-echoic lines” surrounding the thickened GB wall and were intactness which had never been described in malignant GB disease. In addition, in our studies one case with malignant transformation showed destruction of serosal layer on CEUS.

In conclusion, CEUS could increase the visualization of Rokitansky-Aschoff sinuses and is a more accurate means of detection of abnormalities of the GB wall. These CEUS results played important roles in diagnosing adenomyomatousis of the GB. Though the use of CT and MR imaging has also been described in the diagnosis of gallbladder adenomyomatosis, CEUS is the best choice of diagnostic tool in the diagnosis of adenomyomatosis of the GB, as it is real-time, does not involve radiation exposure and is reproducible.

## Limitations and pitfalls

Our study had several limitations. First, the number of was small and only the fundal localized type of adenomyomatosis was assessed. Second, this study showed the value of CEUS in the diagnosis of GB adenomyomatosis, but it was not accurate enough to differentiate the diagnosis of adenomyomatosis from that of early-stage carcinoma or tumor-like lesions of the GB. Of the several lesions causing wall thickness similar to that of the fundal type adenomyomatosis, GB carcinoma is most dreaded. Thus, a further study with a large population including all the types of GB adenomyomatosis and the different disease pathologies of GB is needed.

## Conclusion

In conclusion, CEUS is a better modality that differentiate diagnosis of fundal localized type of gallbladder adenomyomatosis by increasing the visualization of Rokitansky-Aschoff sinuses (RAS). Two “hyper-echoic lines” around the lesion with the small non-enhancement spaces in the arterial phase of CEUS are the characteristic findings.

## Abbreviations

CEUS: Contrast-enhanced ultrasonography
GB: Gallbladder
US: ultrasound
RAS: Rokitansky-Aschoff sinuses
